# Characterization of a Fermented Beverage from Discarded Bread Flour Using Two Commercial Probiotics Starters

**DOI:** 10.3390/foods13060951

**Published:** 2024-03-21

**Authors:** Teresa Sigüenza-Andrés, Javier Mateo, José M. Rodríguez-Nogales, Manuel Gómez, Irma Caro

**Affiliations:** 1 Food Technology Area, College of Agricultural Engineering, University of Valladolid, 34004 Palencia, Spain; teresa.siguenza@uva.es (T.S.-A.); mgpallares@uva.es (M.G.); 2 Department of Food Hygiene and Technology, University of León, Campus de Vegazana s/n, 24071 León, Spain; jmato@unileon.es; 3 Department of Nutrition and Food Science, Faculty of Medicine, University of Valladolid, 47005 Valladolid, Spain; irma.caro@uva.es

**Keywords:** discarded bread flour, sensorial analysis, probiotics, lactic acid bacteria, functional beverage

## Abstract

The aim of this study was to develop a plant-based fermented beverage from discarded bread flour and to analyze its characteristics as a novel functional product. Eight cereal-based probiotic beverages were produced by inoculating discarded bread flour with a monoculture of *Lactobacillus rhamnosus* or a co-culture consisting of lactic acid bacteria and *Bifidobacterium*. Two additional factors, namely, the addition of amylolytic enzymes and matrix desalting, were studied alongside the type of culture. The organic acid content and microbial growth were monitored during fermentation and storage (15 and 21 days). Proximal composition, gamma-aminobutyric acid, and volatile compounds were measured in the final product. Sensory analysis was only conducted on the enzymatically treated samples. The estimated shelf life of the bread beverage was 15 days. The variables studied significantly influenced the amountof organic acids and specific volatile compounds responsible for the aroma of fermented beverages. The beverage produced via co-culturing was preferred by consumers in the sensory test.

## 1. Introduction

Food loss and waste (FLW) is defined as the amount of food and/or associated inedible parts removed from the food supply chain [1] and is a major global problem that affects the sustainability of our planet. FAO [2] estimated that approximately one-third of all food produced worldwide is lost or wasted each year, leading to substantial economic, environmental, and social consequences. Food losses occur before food reaches consumers, while food waste refers to edible food that is discarded at the end of the food chain [3]. The food waste in the last stage of the food chain represents around 30–35% of global FLW [4]. More specifically, bread and bakery products are the most discarded food category after fruit and vegetables.

To tackle the problem of high bread waste, new alternatives for recycling and reusing waste bread, including fermentation, are emerging [5,6]. According to Blandino et al. [7], fermentation is a simple and economical method for improving the nutritional value of cereals, as well as the sensory and functional characteristics of their derivatives. In addition, the beneficial composition of cereals makes them a suitable substrate for lactic fermentation [8]. Several authors reached similar conclusions when using waste bread flour as a substrate for different microorganisms [9,10,11]. Prior to fermentation, the hydrolysis of starch using amylolytic enzymes is necessary in order to convert polysaccharides into fermentable sugars, facilitating bacterial growth, especially under optimized conditions [12].

Within the functional food sector, there is growing demand for fermented beverages [13], and the development of functional plant-based beverages has become an important segment of this market. The rise in the popularity of these products can be attributed to increased consumer interest in functional foods in a context of growing health awareness, changes in food regulations, and the abundance of information regarding the connection between nutrition and health [14]. The organisms most commonly used in the majority of probiotic formulations on the market belong to lactic acid bacteria (LAB) genera, specifically *Lactobacillus*, *Streptococcus*, and *Bifidobacterium* [15,16,17]. LAB, in addition to acting as probiotics, can synthesize gamma-aminobutyric acid (GABA), which plays an important role in the central nervous system [18].

Given the remarkable growth of the market for fermented functional products, it is worthwhile developing new fermented food products, such as fermented beverages, and studying their quality properties, including the content of compounds responsible for their odor and flavor, as well as their sensory acceptability [19]. Another factor influencing the quality of probiotic fermented products is the quantity of probiotics present in a product during processing and storage, as an insufficient dosage at the time of consumption will not provide the expected health benefits [20].

The trend towards vegetarianism and the increase in intolerances and allergies to dairy beverages have positioned plant-based beverages as excellent alternatives with functional properties [21]. So far, many cereal-based fermented functional beverages have been developed using cereals and pseudocereals [22,23]. Both non-functional [24] and functional [25,26] fermented beverages have been developed using waste bread. However, a comprehensive analysis of the characteristics and acceptability of a sustainable probiotic beverage brewed from discarded bread flour has not been undertaken to date.

The aim of this work was to analyze the characteristics of a novel cereal-based probiotic beverage made from discarded bread flour. Eight beverages were prepared using two different starters. This was conducted both with and without an enzymatic process for starch hydrolysis and a desalting pre-treatment. We assessed shelf life, nutritional composition, and the evolution and survival of probiotic microorganisms over 21 days. Additionally, we conducted an analysis of volatile compounds and performed a sensory test with consumers.

## 2. Materials and Methods

### 2.1. Materials

#### 2.1.1. Bread Flour

In order to assist in the production of bread flour, waste wheat bread (1.8% salt, 4% fresh yeast and 55% water per 100 g flour) was donated by the local bakery, La Tahona de Sahagún. Baguettes were dried at room temperature for less than 24 h, and milled in an LM 3100 hammer mill (Perten Instruments, Huddinge, Sweden).

#### 2.1.2. Microorganisms and Enzymes

The commercial starters Nu-trish^®^ LGG^®^ (*Lactobacillus rhamnosus*) and Nu-trish^®^ BY-01 DA (*Bifidobacterium*, *Lactobacillus delbrueckii* subsp. *bulgaricus* and *Streptococcus thermophillus*) were kindly provided by the company CHR Hansen (Hørsholm, Denmark). Starter LGG and starter BY were stored according to the manufacturer’s instructions. Each starter was diluted with 0.01% (*w*/*v*) peptone water (WWR BDH Chemicals, Wayne, WA, USA) plus 0.08% NaCl (Panreac ITW Companies, Barcelona, Spain) at a cell density of 10^7^ CFU/g. Starch hydrolysis was conducted by adding α-amylase (Liquoflow^®^ Yield, Novozyme JSC, Bagsværd, Denmark) and β-glucoamylase (Saczyme^®^ Go, Novozyme, Bagsværd, Denmark).

### 2.2. Methods

#### 2.2.1. Fermentation Procedure and Storage

**Figure 1:**
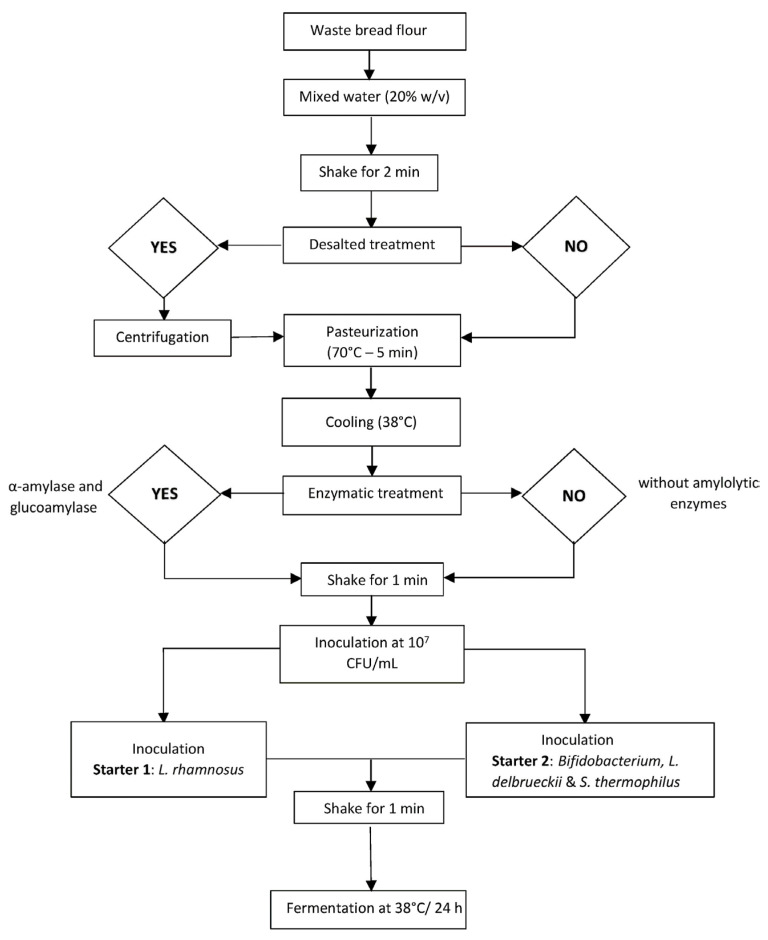
Fermentation procedure by Sigüenza-Andrés et al. [26]. Copyright 2023, Elsevier.

Eight different beverages were developed by combining three study factors: the use of starter BY or LGG, enzymatic addition, and the process of desalting the matrix (Figure 1).

Firstly, bread flour was mixed with sterile water (20% *w*/*v*) by hand. To desalt the flour, the mixture was centrifuged for 5 min at 5000× *g* in a Beckman Coulter J2-HS centrifuge (Pasadena, CA, USA). The supernatant was discarded, and the precipitate was rehydrated again according to the initial proportions (*w*/*v*). Then, the desalted and non-desalted blends were pasteurized at 70 ± 2 °C for 5 min in a thermal bath and cooled in a cold-water bath for about 10 min until reaching 37 °C.

Once all the mixtures had been prepared, the maximum amounts of α-amylase (0.0179 mL/100 g flour) and β-glucoamylase (0.029 mL/100 g flour) recommended by the manufacturer were simultaneously added to half of them, while control samples were kept without the addition of enzymes. Subsequently, an inoculum of 10^7^ CFU/g of bread flour + water taken from each starter was added to four out of eight samples and all were incubated in a water bath at 38 ± 2 °C for 24 h. The fermentation of 900 mL of bread flour and water was carried out in a 1 L glass bottles. Each type of fermentation was performed in duplicate. The final eight beverages included solutions (i) with enzymes and salt (E S), (ii) with enzymes and without salt (E DS), (iii) without enzymes and with salt (NE S), and (iv) without enzymes or salt (NE DS). They were stored for 21 days at 4 °C.

#### 2.2.2. Proximate Composition

The moisture (Moisture Oven Drying, ISO 712:2009 [27]), protein (Kjeldhal method; ISO 20483:2013 [28]), total fat [29], and ash content (ISO 2171:2007 [30]) were measured in the final product. The amount of carbohydrates was calculated based on differences with the other macronutrients. Nutritional composition was measured in duplicate in the final product.

#### 2.2.3. NaCl Content

The NaCl content of the finished products was determined in duplicate according to the research of Carcea et al. [31] using a modified Volhard titration method. Briefly, 10 g of each beverage sample was mixed with 40 mL of distilled water and homogenized for 2 min using a T10 basic Ultraturrax device (IKA-Werke, Staifen, Germany). The resulting mixture was then centrifuged at 500× *g* for 2 min. Then, 25 mL of supernatant was combined with 25 mL of 2% nitric acid (Sigma-Aldrich, St. Louis, MO, USA) to acidify the sample. Autotitration was performed using a chloride-selective electrode (Metrohm, 719 S Tritino, Herisau, Switzerland). The NaCl content was calculated with the following equation:

(1) $$\%\;NaCl=\frac{\left(V\;{AgNO}_{3}\;\times \;C\;{AgNO}_{3}\;\times \;\frac{1L}{1000\;mL}\;\times \;Pm\;NaCl\right)}{V\;sample}$$

where V is volume of sample (mL) and C is the concentration of AgNO_3_ (g/mL).

#### 2.2.4. Determination of Gamma-Aminobutyric Acid (GABA)

Using HPLC-coupled UV-vis for the final product, the determination was performed in duplicate. GABA was extracted according to the method of Erbaş et al. [32]. Briefly, 5 g of bread beverage (at 1, 15, and 21 days) was extracted using 20 mL of 0.01 M KH_2_PO_4_ (PanReac ITW) solution. The extracted product was homogenized at 12,000 rpm using a T-18 Ultraturrax (IKA Labortechnik, Stauten, Germany) and then centrifuged (Sigma M 2-15, Osterode am Harz, Germany) at 3200× *g* for 30 min. The supernatant was frozen at −20 °C until it underwent analysis. Before analysis, the extract was filtered through 0.22 μm pores and 47 mm diameter Whatman PTFE membrane filters (Merck, Darmstadt, Germany).

The chromatography process followed the procedure outlined by Henderson et al. [33]. Then, 0.5 μL of the extract was injected into a model 1200 HPLC chromatograph (Agilent Technologies, Palo Alto, CA, USA) coupled to a UV-vis device. On-line pre-column derivatization was performed with ortho-phthalaldehyde (OPA) and 9-fluorenyl methyl chloroformate (FMOC) for primary and secondary amino groups, respectively. GABA separation utilized a ZORBAX Eclipse AAA column C18 (Agilent, 4.6 mm × 150 mm, particle size 3.5 mm). The mobile phase consisted of NaH_2_PO_4_ (40 mM, pH 7.8; Scharlau, Scharlab S.L., Barcelona, Spain) and acetonitrile/methanol/MiliQ (45:45:10, *v*/*v*/*v*; Fisher Chemical, Pittsburgh, NH, USA and Scharlau, Scharlab S.L., Barcelona, Spain) solutions were used as eluents at 40 °C with a flow rate of 1.5 mL/min. The UV wavelength was 338 nm, and the detection limit was 0.13 g/mL.

#### 2.2.5. Analysis of Volatile Compounds

The production of volatile compounds from bread beverages after 24 h of fermentation was tested using solid-phase microextraction (SPME) coupled with gas chromatography/mass spectrometry. Following the procedure described by Soto et al. [34], the SPME extraction of headspace volatile compounds was carried out in duplicate using 6 g of bread beverage. The chromatographic separation and identification of the volatile compounds were performed according to the procedure described by Carballo et al. [35].

#### 2.2.6. Consumer Acceptance

Only enzyme-treated samples were evaluated to assess their sensory qualities due to their liquid texture, as the other samples had a puree texture that was not suitable for use in beverages. The sensory characteristics of each probiotic beverage were evaluated by 95 volunteers from the College of Agricultural Engineering (Palencia, Spain), aged between 18 and 64 years. The consumer sensory assessments were conducted in individual booths within a tasting room, adhering to the guidelines outlined in the ISO 8589 Standard [36].

The drinks were tasted within 48 h of fermentation. Before consumption, they were homogenized in a blender (Thermomix Vorwerk, Wuppertal, Germany) for 10 min. Additionally, 15 g/L sugar (Azucarera, Madrid, Spain) and 0.55 g/L cinnamon (Carmencita, Alicante, Spain) were added to each beverage. Fifteen mL of the four fermented beverages was dispensed into clear plastic cups. These were appropriately coded and presented to each consumer for the evaluation of sensory attributes such as appearance, taste, texture, odor, and overall acceptability. The hedonic scale ranged from 1 (‘I don’t like it at all’) to 9 (‘I like it very much’). Furthermore, comments were collected from the consumers [37].

#### 2.2.7. Microbial Determination

At the end of the fermentation period, after 15 and 21 days, 10 g of each beverage was taken with a sterile pipette and mixed with 90 mL of peptone water (0.01% peptone, 0.85% NaCl) in a 100 mL volumetric flask. The mixture was shaken for 2 min and then diluted 10-fold with sterile peptone water.

The drop method [38] was used for bacterial counting. *L. rhamnosus* LGG (starter LGG) and *S. thermophilus* were counted on MRS agar (Agar Man, Rogosa and Sharpe, VWR BDH) after 48 h of incubation and on M17 agar (Oxoid CM0785, Basingstoke, UK) supplemented with 10% lactose monohydrate (Merck) after 24 h of incubation, respectively. Incubation was carried out in a bacteriological oven (Giralt S.A, Barcelona, Spain) at 37 ± 2 °C.

*Bifidobacterium* was cultured on MRS agar following the method by Sigüenza-Andrés et al. [26] and incubated in an anaerobic jar (Oxoid) for 72 h at 37 °C. Finally, *L*. *delbrueckii subp bulgaricus* was counted on a double-layer MRS agar after the inoculation of 1 mL of the appropriate dilution and incubation at 42 °C for 48 h. The counts were expressed as the logarithm of colony-forming units (Log-CFU). Curve modeling and growth parameters were calculated from microbial kinetics using the web version of DMFit (Institute of Food Research, Norwich, UK; [39]). The mean values of replicates were obtained from two independent fermentations.

#### 2.2.8. Analysis of Organic Acids

The extraction procedure was identical to that described in the GABA section. Twenty μL of the extract was injected into the HPLC chromatograph (Agilent 1260-Infinity II) using a Coregel 87H3 column (7.8 mm internal diameter and 300 mm of length) with a UV detector set to a wavelength of 210 nm. For the mobile phase, a 0.008 N NH_2_SO_4_ solution (Honeywell Fluka ^TM^, Steinheim, Germany) was used at 35 °C with a flow rate of 0.6 mL/min.

Organic acid standards (Sigma-Aldrich) were prepared in the mobile phase, and a calibration line was prepared for each of them. The peaks were verified by adding standard solutions of organic acids to several samples. The results were calculated on a dry-weight basis, and the detection limits were as follows: acetic acid, 1.31; lactic acid, 0.25; propionic acid, 3.96; butyric acid, 2.77; pyruvic acid, 1.65; and malic acid, 2.68 mg/L. Organics acids were also analyzed after 1, 15, and 21 days.

#### 2.2.9. Analysis of Carbohydrates

Carbohydrate analysis was performed in duplicate using high-performance anion-exchange chromatography with pulsed amperometric detection (HPAEC-PAD). We extracted sugars from bread beverages and implemented our instrumental conditions according to the specifications of Sigüenza-Andrés et al. [26]. A 1:20 dilution of all the samples was performed due to their high glucose concentrations. Each analysis was carried out on a Metrohm system (Metrohm, Herisau, Switzerland) and the detection limits were as follows: glucose, 13.939; isomaltose, 0.422 and maltose, 1.447 mg/L. Carbohydrates were measured at the end of fermentation and after 15 and 21 days.

#### 2.2.10. Statistical Analysis

Analysis of variance (ANOVA) was performed for the other experiments using STATGRAPHICS Centurion XV (StatPoint Technologies, Inc., Warrenton, VA, USA). Values of *p* < 0.05 were considered statistically significant based on Tukey’s least-significant differences.

## 3. Results and Discussion

### 3.1. Macronutrients

The proximate compositions of the beverages are shown in Table 1. Considering that milk has 88% water in its composition, the moisture content obtained shows normal values for fermented beverages. No significant differences in moisture content were observed as the humidity was kept constant (*p* = 0.05). Regarding nitrogen content, none of the examined factors (type of starter, enzymatic treatment, and desalting) resulted in statistically significant differences among the beverages. The protein content of the bread flour was reported to be approximately 10 g/100 g, whereas the content of the beverages was five times lower in percentage terms (20% *w*/*v*). This content was comparable with the values reported for commercial plant-based yogurts derived from cashews and almonds (2.00–2.30 g/100 g), but was lower than that observed in soy and dairy products (4.00 and 5.10 g/100 g), respectively [40]. The low protein content may give a low buffering capacity to the beverages, which could permit them to reach values close to or less than pH 3 [26].

No significant differences were found among samples concerning carbohydrate content (*p* = 0.21). This content was slightly lower than that obtained by Pontonio et al. [41] in a yogurt-style snack made of rice, lentils, and chickpea flour. As expected, significant differences were found in NaCl content between samples with and without salt, since the desalting process produced a reduction of approximately 50% in the salt content of the beverages. Similar NaCl values to those of our salted samples were reported for oat-based products (0.22 g/100 g) [42].

Concerning ash content, the salted samples had a significantly higher quantity of ash (*p* = 0.0002). This could be explained by the desalting process, where certain minerals were discarded within the supernatant. Intermediate values were obtained by Bernat et al. [43] compared to those observed in these beverages, and they found an ash content of 0.33 g/mL in a fermented almond drink.

The highest concentration of GABA (*p* = 0.00) was found in salted samples, indicating the positive influence of salt on its production. This fact could have three explanations. Firstly, LAB may use sodium as a transmembrane transporter to introduce glucose into cells via a sympathetic transport pathway [44]. Secondly, LAB might employ glucose by the glycolysis pathway and produce pyruvic acid, and this subsequent increase in the metabolite could contribute to the accumulation of GABA. In the BY starter, this phenomenon can be attributed to the co-fermentation of *L. delbrueckii* and *S. thermophilus*, which is known to elevate GABA levels [18]. Thirdly, the production of lactic acid via glycolysis affects the physiological activities of LAB. Under acidic conditions, LAB have evolved several acid resistance systems in order to maintain cell viability. One of these systems is the glutamate-dependent system, which requires the presence of glutamate as a substrate. Glutamate-dependent systems consume intracellular protons by combining them with internalized glutamate to form GABA. This exchange of compounds increases the intracellular pH values. In this study, GABA production was dependent on the amount of lactic acid produced. In fact, culture samples without enzymes and without salt showed the lowest levels of lactic acid and consequently produced the least amount of GABA [45].

As GABA is an important bioactive component that functions as an inhibitory neurotransmitter in the brain, higher contents are valuable in fermented products. The GABA content in our beverages was higher than the amount reported in a novel yogurt-style snack made with LAB and legume flour (0.01 g/100 g) [41], even when comparing the beverages with the lowest GABA content (LGG NE DS, BY E DS and BY NE DS). Dose-dependent beneficial effects were observed in the range of 1–1000 µg [46]. All the fat values were below the detection limit. This is logical since the bread flour used did not contain any fat. These beverages have good properties, as their fat percentage is lower than that of skimmed dairy products.

### 3.2. Volatile Compounds

The content of volatile compounds in the beverages fermented for 24 h is shown in Table 2. A total of 19 compounds were positively identified and grouped into selected chemical families. Among the identified compounds, acetic acid, ethanol, butane-2,3-dione (diacetyl), pentane-2,3-dione and 3-hydroxybutane-2-one (acetoin), as well as the branched-chain alcohols and aldehydes, could have been produced from the metabolism of LAB [23,46]. The remaining compounds, namely hexanol, pentanal, hexanal and hydrocarbons, would have originated mainly from the auto-oxidation of fatty acids [47,48]. According to previous studies, acetic acid and ethanol are compounds which are characteristically produced during fermentation by *L. rhamnosus* and thermophilic *Streptococcus* [49,50], and acetic acid is also produced by *Bifidobacterium* due to glucose fermentation via the fructose-6-phosphate shunt or bifid pathway [51]. *Lactobacillus rhamnosus* is considered a facultative heterofermentative bacterium, meaning that it can utilize different pathways for fermentation depending on the available substrates. It primarily produces lactic acid from glucose through the glycolysis pathway. However, it can also ferment pentoses via the 6-phosphoteketolase (6-PK) pathway, resulting in the production of lactic acid, acetic acid, and ethanol [52,53]. This explains why ethanol is present in beverages containing *Lactobacillus rhamnosus* in this study.

Overall, the headspace of the BY beverages contained more acetic acid than that of the LGG beverages (Table 2). This difference is consistent with the results of the study by Bujna et al. [54], who reported a higher acetic acid content in beverages fermented with mixed LAB and *Bifidobacterium* cultures (27–48 mM) compared to those fermented with LAB monocultures (18–30 mM).

In the BY beverages, the use of enzymes tended to increase acetic acid levels, with significant differences being found between BY E S a BY NE S. This could be related to the higher availability of fermentable sugar in the enzyme-treated beverages. In addition, acetic acid levels were significantly greater in both non-desalted (S) beverages than in those previously desalted (DS). This suggests that glucose transport pathway may have been enhanced by the presence of sodium, as mentioned above. Meanwhile, in the LGG beverages, acetic acid levels were only detected in the E S and E DS beverages (both with high a availability of fermentable sugar). Despite these differences, acetic acid is not expected to have a distinctive impact on the taste of the produced fermented beverages as their odor thresholds are relatively high compared to the other end products of LAB fermentation [54,55].

The acetaldehyde concentration was higher in BY beverages than in LGG, with no clear effect observed from the presence of enzymes or salts. The low amount of acetaldehyde can be explained by the fact that LGG growth under low O_2_ concentrations canactive ethanolic fermentation. In this pathway, acetaldehyde is converted into ethanol by the enzyme alcohol dehydrogenase [56]. Thus, the BY fermentation process would have a higher capacity to synthesize acetaldehyde than the LGG culture. Both, *L. delbrueckii* and *S. thermophilus* are able to produce acetaldehyde, although its production appears to be strain-specific [55]. Acetaldehyde, with green apple or nutty flavor notes and a low odor threshold, is widely associated with the typical aroma of yoghurt [55]. Salmerón et al. [19] reported that high levels of acetaldehyde could lead to greater acceptance of a malt-based beverage.

In this study, butane-2,3-dione (diacetyl) was detected in all the fermented beverages, with no clear differences in levels between LGG and BY beverages. However, acetoin was only detected in those beverages with enzymes and no desalting (E S). Moreover, diacetyl levels tended to be higher in enzyme pre-treated beverages, especially in LGG beverages, suggesting that glucose availability may favor diacetyl formation. The presence of these two C4 compounds in LAB-fermented beverages is derived from 2-acetolactate, which, in turn, is formed from citric acid through various pathways of glycolytic metabolism. Previous studies have shown that *L. rhamnosus* and *S. thermophilus* have high potential to produce diacetyl [50,57], while *L. delbrueckii* is also able to produce diacetyl, albeit at very low levels [58]. Diacetyl and acetoin impart creamy and buttery aroma notes in dairy products fermented by LAB.

Pentane-2,3-dione was only detected in the BY beverages (in all of them except for BY NE DS; Table 2). The direct precursor of pentane-2,3-dione is 2-aceto-hydroxybutyrate, which, like diacetyl, is formed from acetaldehyde [59]. According to Masiá et al. [60], Streptococcus normally produces pentane-2,3-dione and its lower growth in the BY NE DS sample may explain the absence of this compound in that beverage. Wang et al. [44] also detected this compound in a soy- and oat-based yogurt, where its levels were found to be related to the yogurt flavor, with an odor described as buttery, vanillaesque, and mild. It is considered to be a key flavor compound in yogurt [59].

The sum of the levels of the branched-chain aldehydes compounds did not exhibit clear differences among beverages. Individually, the levels of 2-methylpropanal and 2-methylbutanal tended to be higher in BY than in LGG, although for the latter, no significant differences were observed between treatments (Table 2). The branched-chain aldehydes and alcohols detected in this study were formed by LAB in fermented dairy products from leucine, isoleucine, and valine after conversion into their respective α-keto-acids [23,61]. These compounds have been identified as potent odorant compounds (malty scent for aldehydes and fruity scent for alcohols), i.e., yoghurt- and cereal-based liquid fermentations.

In this study, hexanol, pentanal, or hexanal was detected in six of the eight fermented beverages, with hexanal being the most abundant compound, and the LGG E S beverage exhibited the highest hexanal levels. The presence of hexanal in LAB-fermented beverages was influenced by factors related to fatty acid oxidation, with one of these factors being the lipolytic activity of LAB. Moreover, Bifidobacterium is able to catabolize hexanal and pentanal [62], potentially contributing to reduced levels in fermented beverages containing these bacteria. Differences in hexanal content among fermented beverages can have an impact on its flavor, as hexanal has a low odor threshold (green or cut grass) [55]. Finally, straight-chain hydrocarbons were also detected in the beverages, and their levels showed no significant differences between treatments (Table 2) (*p* = 0.520). The expected impact of these compounds on flavor is low due to their high odor threshold [48].

### 3.3. Sensory Analysis

**Figure 2:**
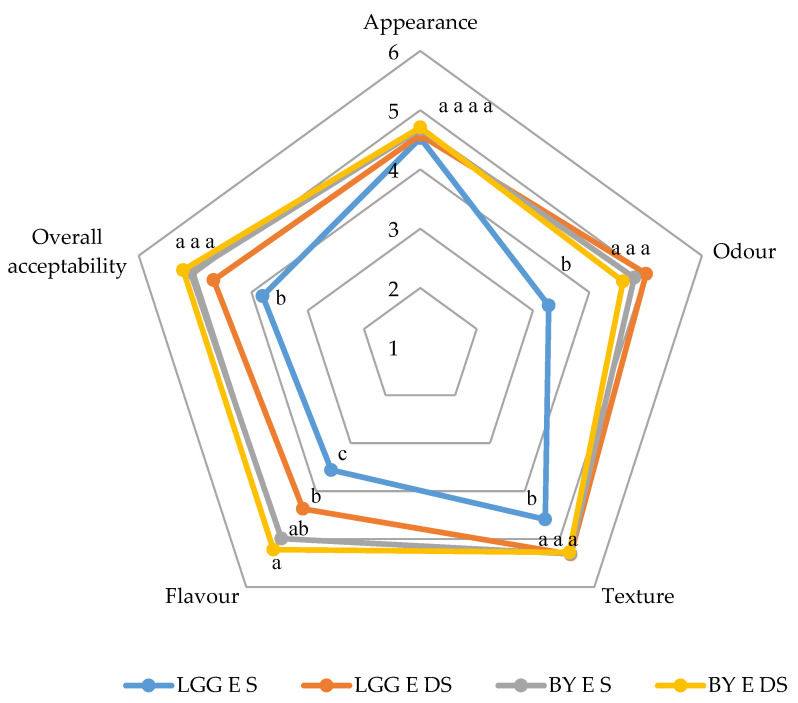
Consumer analysis of enzymatically hydrolyzed beverages after fermentation. LGG: Nu-trish^®^ LGG^®^; BY Nu-trish^®^ BY-01 DA; S and DS: with salt and desalted; E: with enzymes. Data are expressed as mean ± standard deviation (n = 4). Values of the same attribute with different letters differ significantly (Tukey test; *p* < 0.05). Acceptability was evaluated using a structured hedonic scale of 9 points, from 1 (‘I don’t like it at all’) to 9 (‘I like it very much’).

The enzyme-treated beverages were selected for consumer sensory analysis due to their less thickened textures, resulting from enzymatic hydrolysis; the others were not considered. The results obtained from the evaluation of the four chosen beverages are shown in Figure 2. When analyzing the measured hedonic attributes, significant differences were detected between the LGG E S sample and the others, except in terms of appearance. The lack of differences in appearance suggests that all the four beverages had similar characteristic brownish colors provided by the bread flour.

In terms of texture, the LGG E S sample appeared to have a mash-like texture, which would be perceived as an undesirable characteristic. In terms of odor and flavor, this sample was also negatively highlighted by consumers, who indicated in their comments that it had a pronounced sour smell and the worst flavor among the samples tested. The reduced odor and flavor scores of LGG E S are consistent with the higher levels of lactic acid formation during fermentation, as described below (Section 3.4). The lower scores in flavor for LGG E S may be related to its higher hexanal levels, as mentioned above (Table 2).

Overall acceptability seemed to follow the behaviour of flavor and texture. As mentioned above, the BY beverages had the highest acceptability (>5 points over 9), and both were assessed as having generally mild odors and flavors. The quantitative acceptability ratings of these beverages were similar to those obtained using buckwheat bread leftovers, especially for samples made without fungal pre-treatment. Salmerón et al. [19] obtained worse results in beverages fermented with *L. reuteri* (2.95–3.18 out of 9) and *L. acidophilus* (2.71–3.23 out of 9) using barley, malt, and oat substrates, respectively. Conversely, comparable results were found in beverages formulated with *L. plantarum* (3.58–5.33 out of 9). Further work is needed to improve the evaluation of beverages, focusing in particular on flavor because of its relevant role in the final levels of acceptance.

### 3.4. Microbial Growth

Table 3 shows the counts of each bacterium in each starter throughout the storage period. At the end of the fermentation, *Bifidobacterium* reached the highest counts in the BY starter. Conversely, Streptococci and Lactobacilli presented the lowest counts.

However, these individual values were surpassed by those of the LGG starter, which had slightly higher counts at 24 h. Although *S. thermophilus* and *L. delbrueckii* had a symbiotic relationship, the combination of the three microorganisms and the fermentation conditions may have had a negative impact on their own growth. Microbial counts were slightly lower than those found by Voss et al. [63] in a soy beverage, standing at 9.17 and 9.31 Log CFU/mL for *Bifidobacterium* and *Lactobacillus rhamnosus*, respectively.

Observing evolution throughout storage, all microorganisms except for LGG E S suffered a decrease at 21 days, although this decline was much more pronounced for *L. delbrueckii* and *Bifidobacterium*. In fact, the latter kept growing during the first two weeks of storage and then fell critically in salted samples (below the detection limit) and by around one logarithmic (Log) unit in desalted beverages, making the presence of salt a limiting factor in survival. According to these results and Spanish regulations [64], the shelf life of beverages made with the BY starter is limited by the growth of *Bifidobacterium*, which reduces their shelf lives to 15 days.

Regarding *L. rhamnosus*, it showed a growth tendency in the first two weeks, but, after 15 days, its growth remained almost stable. Moreover, the LGG starter was neither affected by the enzymatic nor the desalted treatment during the storage at 15 and 21 days, as no significant differences were found between the four LGG beverages. In the case of BY, the desalting treatment reduced the counts and its combination without enzymes had a negative effect, particularly on *L. delbrueckii* and *Streptococcus* growth.

The survival of probiotics in food matrices, such as non-dairy products, is one of the most important aspects to consider, and their viability depends on factors such as pH, storage temperature, and the presence of competing microbes and inhibitors, p.e. NaCl or oxygen concentration [63]. The higher NaCl concentration detected in the beverages produced without the desalination pre-treatment (see Table 1) might be the primary cause for the diminished viability of *Bifidobacterium* (<100 cells/mL) after 21 days. Moreover, interactions may arise in the mixed culture, such as with *L. delbrueckii*, affecting *Bifidobacterium* in two ways. Firstly, *L. delbrueckii* strains might increase bifidobacterial growth due their proteolytic activity, increasing valine, glycine, and histidine availability [65]. Secondly, Lactobacilli produce hydrogen peroxide when oxygen is present, with inhibitory effects [66]. In this study, more counts of *L. delbrueckii* were found at 21 days in beverages with salt, but the *Bifidobacterium* counts were lower (below the detection limit). These counts could indicate that the negative impact of *Lactobacillus* prevails over its positive effect, or even that the accumulation of fermentation metabolites combined with salt has a significant negative effect on *Bifidobacterium*.

The ideal level of probiotic microorganisms required to benefit health depends on the strains and the product used, although it must be between 10^6^ and 10^8^ viable cells per mL or g of product [67]. In this study, the fermented plant-based beverage with LGG and *Bifidobacterium* (BY) had higher levels than these at the end of the fermentation.

### 3.5. Determination of Organic Acids

Table 4 shows the evolution of the concentration of different organic acids in the beverages studied during storage. Both starters mainly produced lactic acid, but they also released other organic acids such as acetic, citric, and propionic acids. After 24 h of fermentation, BY E S and LGG E S obtained the maximum amount of lactic acid in each starter, respectively. The results showed that there was no clear effect of salt in any of the beverages, but that the enzymatic treatment could have enhanced its production. This was because the greater the availability of glucose is, the higher lactic acid yield will be [68], as both metabolisms are related (see Table A1). A higher lactic acid content was found in a fermented beverage based on sprouted oat flour (1.63 g lactic acid/100 g) produced by *L. plantarum* [22]. This difference could be due to the use of different substrates, as the sprouted oat flour, rich in enzymes, seemed to be more suitable for the growth of *L. plantarum* and yielded more lactic acid.

As at 24 h, the absence of enzymes and the desalting process negatively affected the production of lactic acid in both starters during storage (15 and 21 days). This fact could be related to the growth of starters, as *L. delbrueckii* and *L. rhamnosus* showed lower counts in the BY NE DS and LGG NE DS samples, respectively. On the contrary, the fermented bread samples containing salt and enzymes reached the highest lactic acid concentration. In this study, post-acidification depended on the presence of enzymes and mainly on the type of starter used during fermentation, since the ES sample of the BY starter, composed of three bacteria, produced half the lactic acid compared to LGG E S at 21 days. A similar trend was observed by Helland et al. [69] in water-based puddings, where they obtained much more lactic acid with *L. rhamnosus LGG* than with a combination of *B. animalis BB12* + *L. acidophilus La5*. This discrepancy may be attributed to the lower growth of *L. acidophilus* in the latter study or *L. delbrueckii* in our case (Table 3).

The production of organic acids, mainly lactic acid, is a key role of LABs in fermented food production. These acids exhibit bacteriostatic activity, improving food safety by restricting the proliferation of undesirable microorganisms [70]. However, the production of large quantities of those organic acids reduces the viability of *Bifidobacterium* throughout the shelf life of probiotic products due to acid stress during the fermented processing [71].

Acetic acid was the second most produced acid, with similar values found in all the beverages. Higher amounts of acetic acid have been reported when using alternative plant-based substrates, with values ranging from 0.90 g/100g [19] to between 1.1 g to 2.5 g/100 g [22]. No significant differences were observed in the evolution of acetic acid content during storage in either starter (*p* > 0.05). Moreover, there was no clear evidence of how the variables affected acetic acid production, as significant differences were only found at 24 h, and the differences were so minimal that a clear trend there was not apparent. However, the formation of acetic acid in BY samples might be explained by the high growth of *Bifidobacterium* with both enzymes and salt at this time, in contrast to samples grown with enzymes but without salt. It is known that *Bifidobacterium* produces acetic acid from glucose through bifid metabolism and that its production is influenced by matrix components. According to Nguyen et al. [72], *B. lactis* was able to produce more acetic acid than lactic acid (0.138 vs. 0.062 g/100 g). However, in this study, the acetic acid was released in lower concentrations, even in BY samples that contained *Bifidobacterium*.

In addition, the results showed differences between acetic acid, measured as a volatile compound via the headspace, and acetic acid content measured via HPLC.

In general terms, the BY starter formed more citric acid than *L. rhamnosus* during the fermentation and storage periods, with significant differences observed between starters after 24 h and 15 days (*p* < 0.05). No significant differences were noted during the storage of the beverages, and there was no specific effect of salt and enzyme factors (*p* > 0.05). Within BY samples, those with salt showed a significant decrease in citric acid from the end of fermentation to after three weeks of storage, while no differences were observed between desalted samples. Erbaş et al. [32] reported that the decrease in citric acid content during fermentation could be attributed to its utilization as a substrate in secondary reactions during the fermentation process.

During propionic acid production, no clear effects were observed for the three factors individually. Nevertheless, the combination of the desalting process and the absence of enzymes (NE DS) appears to enhance the production of propionic acid in the BY starter, reaching peak values at all three time points.

Butyric acid was produced in very small quantities. In general, no differences were observed between the beverages, whether by the type of starter, enzymatic treatment, or desalting. This was noted at 1, 15, and 21 days.

## 4. Conclusions

The present study demonstrates the viability of utilizing discarded bread flour as a substrate for various strains of LAB and *Bifidobacterium* in order to develop a novel plant-based probiotic beverage. The concentrations of organic acids in the final product, particularly of acetic and lactic acid, were found to be sufficiently high to ensure a pH value that guarantees the microbiological safety of the product. Additionally, the bread-based beverage was successfully fermented and remained stable for 15 days, despite a slight decrease in *L. delbrueckii*. Among the end products of fermentation, the presence of GABA in concentrations high enough to produce beneficial effects on the organism is noteworthy. The beverages also exhibited the presence of volatile compounds, contributing to a typical yoghurt aroma. Samples fermented with the BY co-culture were more accepted by consumers, achieving favorable ratings for texture, flavor, and overall score.

This innovative functional beverage not only acts as a sustainable and eco-friendly solution for reducing bread waste, but also provides a rich source of probiotics, enhancing gut health, immune modulation, and overall well-being. Moreover, bread flour is a reliable source of essential nutrients and, through the fermentation process, it promotes the synthesis of bioactive compounds with potential health benefits. Further research is required to maintain the microbial load and to improve the shelf life of the final product throughout the storage, as well as to refine the sensory characteristics and physical stability of the beverages.

## Figures and Tables

**Table 1:** Proximate composition and NaCl and gamma-aminobutyric acid (GABA) contents in the beverages after 24 h of fermentation.

	LGG				BY			
	E S	NE S	E DS	NE DS	E S	NE S	E DS	NE DS
Moisture (%)	81.77 ± 0.13	80.95 ± 0.28	83.84 ± 1.09	83.30 ± 0.09	81.71 ± 0.25	82.59 ± 0.19	83.66 ± 0.28	83.72 ± 2.29
Protein (%)	2.34 ± 0.12	2.38 ± 0.03	2.03 ± 0.02	1.93 ± 0.16	2.38 ± 0.01	2.17 ± 0.00	2.06 ± 0.19	1.89 ± 0.38
Carbohydrates (%)	15.20 ± 0.05	15.95 ± 0.39	13.69 ± 1.06	14.48 ± 0.09	15.15 ± 0.16	14.55 ± 0.23	13.97 ± 0.09	14.09 ± 1.90
NaCl (%)	0.22 ± 0.00 b	0.23 ± 0.01 ab	0.13 ± 0.02 c	0.11 ± 0.01 c	0.23 ± 0.00 b	0.27 ± 0.01 a	0.12 ± 0.00 c	0.12 ± 0.01 c
Ash (%)	0.48 ± 0.05 ab	0.49 ± 0.08 ab	0.31 ± 0.00 bc	0.18 ± 0.02 c	0.54 ± 0.08 a	0.43 ± 0.03 ab	0.19 ± 0.01 c	0.19 ± 0.00 c
GABA (mg/100 g)	0.26 ± 0.10 a	0.27 ± 0.02 a	0.18 ± 0.14 ab	0.05 ± 0.01 bc	0.25 ± 0.03 a	0.18 ± 0.02 ab	0.02 ± 0.01 c	0.03 ± 0.02 c

LGG: Nu-trish^®^ LGG; BY: Nu-trish^®^ BY-01 DA; E and NE: beverages with and without the addition of enzymes, respectively; S and DS: beverages with salt and desalted, respectively. All fat values were below the detection limit (0.083% Soxhlet). Carbohydrates were calculated based on difference. Data are expressed as mean ± standard deviation (n = 8). Values in the same row with different letters differ significantly (Tukey test; *p* < 0.05). A lack of letters means no significant differences.

**Table 2:** Volatile compounds extracted from headspace of the beverages, expressed as peak area units × 10^−6^, after a 24 h fermentation period.

	LGG				BY			
	E S	NE S	E DS	NE DS	E S	NE S	E DS	NE DS
2-Carbon compounds								
Acetic acid	9.1 ± 12.8 c	-	3.4 ± 4.8 c	-	1528.6 ± 232.0 a	772.0 ± 17.0 b	255.3 ± 117.9 c	62.7 ± 50.0 c
Ethanol	19.0 ± 12.6	60.9 ± 27.4	14.9 ± 4.4	53.2 ± 35.8	13.4 ± 4.2	33.0 ± 2.7	22.2 ± 7.1	40.9 ± 14.1
Acetaldehyde	2.8 ± 3.9 c	0.6 ± 0.9 c	2.7 ± 3.8 c	2.1 ± 0.1 c	29.0 ± 3.0 ab	32.7 ± 2.0 a	23.4 ± 0.4 ab	19.8 ± 3.2 b
*Partial sum*	30.8 ± 4.1 c	61.5 ± 28.2 c	21.0 ± 4.3 c	55.3 ± 35.9 c	1570.9 ± 232.5 a	837.6 ± 16.3 b	300.8 ± 110.2 c	123.2 ± 67.2 c
Ketones								
Butane-2,3-dione	871.1 ± 270.6 a	338.0 ± 122.8 bc	699.0 ± 62.3 ab	192.5 ± 0.9 c	513.2 ± 38.7 abc	301.2 ± 28.1 bc	489.5 ± 30.1 abc	145.4 ± 46.1 c
Pentane-2,3-dione	-	-	-	-	20.9 ± 1.7	10.1 ± 14.3	5.8 ± 8.2	-
3-Hydroxybutan-2-one	25.3 ± 8.7	-	-	-	3.6 ± 0.8	-	-	-
Partial sum	896.4 ± 279.6 a	338.0 ± 122.8 bc	699.0 ± 62.3 ab	192.5 ± 0.9 c	537.6 ± 41.2 abc	311.3 ± 42.4 bc	495.3 ± 21.9 abc	145.4 ± 46.1 c
Branched-chain alcohols and aldehydes								
2-Methylpropan-1-ol	6.8 ± 0.4	10.2 ± 4.6	5.2 ± 1.1	5.0 ± 7.1	5.1 ± 2.3	9.9 ± 0.2	-	-
3-Methylbutan-1-ol	47.0 ± 34.7	-	-	-	17.4 ± 0.2	-	37.5 ± 13.1	4.1 ± 5.7
2-Methylpropanal	0.5 ± 0.6 b	3.3 ± 2.7 b	2.7 ± 0.4 b	4.9 ± 2.1 ab	8.3 ± 4.8 ab	15.8 ± 3.8 a	9.4 ± 3.5 ab	7.1 ± 2.1 ab
3-Methylbutanal	-	13.8 ± 4.1	1.4 ± 2.0	10.7 ± 6.0	2.0 ± 2.8	9.0 ± 0.1	4.9 ± 6.9	8.3 ± 1.3
2-Methylbutanal	-	1.5 ± 2.1	-	4.4 ± 1.8	10.3 ± 8.0	16.8 ± 2.6	7.8 ± 4.0	10.3 ± 2.1
Partial sum	54.3 ± 33.7	28.8 ± 13.4	9.3 ± 1.3	25.0 ± 17.0	43.0 ± 1.9	51.4 ± 6.2	59.5 ± 19.6	29.8 ± 11.2
Straight-chain 5- to 6-carbon alcohols and aldehydes								
Hexanol	-	-	5.1 ± 7.2	-	5.8 ± 8.2	-	5.1 ± 7.1	-
Pentanal	-	-	20.0 ± 12.2	-	-	-	-	10.3 ± 14.5
Hexanal	128.3 ± 3.8 a	-	32.4 ± 45.8 b	-	10.4 ± 4.7 b	5.4 ± 7.6 b	-	17.2 ± 24.3 b
Partial sum	128.3 ± 3.8 a	-	57.4 ± 50.7 ab	-	16.2 ± 12.9 b	5.4 ± 7.6 b	5.1 ± 7.1 b	27.5 ± 38.8 ab
Hydrocarbons								
Pentane	6.0 ± 2.7	16.6 ± 8.0	5.7 ± 1.0	16.1 ± 3.0	7.1 ± 1.7	8.1 ± 1.8	5.5 ± 1.0	26.3 ± 20.2
Hexane	15.3 ± 5.8	32.2 ± 18.0	20.7 ± 2.3	37.5 ± 23.1	35.1 ± 24.4	14.6 ± 2.7	18.0 ± 1.6	22.1 ± 1.3
2,2,4-Trimethylpentane	13.2 ± 9.0	12.8 ± 11.5	12.7 ± 1.1	12.6 ± 7.7	4.6 ± 6.4	7.8 ± 2.1	15.2 ± 4.4	15.5 ± 0.6
Octane	16.8 ± 1.6	17.0 ± 8.9	20.6 ± 4.3	-	-	-	2.8 ± 4.0	10.3 ± 8.2
Decane	105.9 ± 75.6	143.4 ± 23.3	316.4 ± 22.8	265.2 ± 106.8	135.6 ± 100.5	741.7 ± 813.9	394.2 ± 80.5	431.1 ± 53.2
Partial sum	157.1 ± 68.3	221.9 ± 0.1	376.1 ± 29.2	331.3 ± 94.4	182.2 ± 80.8	772.2 ± 812.5	435.6 ± 80.3	505.2 ± 79.8
Total sum	1266.8 ± 389.1 ab	650.1 ± 81.2 b	1162.7 ± 43.7 ab	604.0 ± 112.3 b	2349.8 ± 365.5 a	1977.8 ± 869.7 ab	1296.2 ± 224.8 b	831.0 ± 220.6 b

- Content below the detection limit (0.3 × 10^6^ peak area units). LGG: Nu-trish^®^ LGG; BY: Nu-trish^®^ BY-01 DA; E and NE: with and without addition of enzymes; S and DS: with salt and desalting. Data are expressed as mean ± standard deviation (n = 8). Values in the same row with different letters differ significantly (Tukey test; *p* < 0.05). No letters mean no significant differences.

**Table 3:** Count of microorganisms of LGG and BY starters in the beverages after fermentation and during storage. Factors: Nu-trish^®^ LGG^®^ (LGG), Nu-trish^®^ BY-01 DA (BY), with (E) and without (NE) addition of enzymes, with (S) and without (DS) salt.

Starter	Log CFU/g t1d	Log CFU/g t15d	Log CFU/g t21d
*Bifidobacterium* BY E S	7.97 ± 0.09 a	9.78 ± 0.34 b	-
*Bifidobacterium* BY E DS	7.48 ± 0.07 b	6.90 ± 0.14 d	6.73 ± 0.75
*Bifidobacterium* BY NE S	7.97 ± 0.14 a	10.35 ± 0.05 a	-
*Bifidobacterium* BY NE DS	7.96 ± 0.03 a	8.68 ± 0.03 c	6.75 ± 0.16
*Lactobacillus* BY E S	4.67 ± 0.21 b	4.36 ± 0.88	3.74 ± 0.00
*Lactobacillus* BY E DS	4.21 ± 0.07 c	1.60 ± 0.58	1.35 ± 0.56
*Lactobacillus* BY NE S	5.33 ± 0.11 a	4.73 ± 0.30	3.16 ± 0.56
*Lactobacillus* BY NE DS	3.28 ± 0.04 d	2.79 ± 1.44	1.83 ± 0.00
*Streptococcus* BY E S	7.20 ± 0.76 a	6.94 ± 0.13	6.26 ± 0.10
*Streptococcus* BY E DS	6.22 ± 0.01 ab	6.28 ± 0.06	6.08 ± 0.35
*Streptococcus* BY NE S	6.64 ± 0.00 a	6.21 ± 1.33	6.48 ± 1.95
*Streptococcus* BY NE DS	5.30 ± 0.13 b	4.30 ± 0.13	4.38 ± 0.03
*Lactobacillus* LGG E S	8.11 ± 0.02 b	8.91 ± 0.08	8.69 ± 0.30
*Lactobacillus* LGG E DS	8.63 ± 0.07 a	8.36 ± 0.27	7.93 ± 0.69
*Lactobacillus* LGG NE S	8.45 ± 0.22 a	8.06 ± 0.01	7.93 ± 0.05
*Lactobacillus* LGG NE DS	7.95 ± 0.01 b	7.75 ± 0.13	7.79 ± 0.00

- Counts below the detection limit (10^2^ CFU/g). t_1d,_ t_15d_, and t_21d_ are the microorganisms counts at 1, 15 and 21 days, respectively (log CFU/g). Data are expressed as mean ± standard deviation (n = 8). Values in the same column of the same frame with different letters differ significantly (Tukey test; *p* < 0.05). No letters mean no significant differences.

**Table 4:** Contents and evolution of organic acids in the beverages at 1, 15, and 21 days of storage.

Fermentation Time (d)	Sample	Lactic (g per 100 g)	Acetic (g per 100 g)	Citric (g per 100 g)	Propionic (g per 100 g)	Butiric (g per 100 g)
1	LGG E S	0.7224 ± 0.0249 a	0.0316 ± 0.0015 c	0.0037 ± 0.0018 c	0.0039 ± 0.0039 ab	-
	LGG NE S	0.2510 ± 0.0454 c	0.0362 ± 0.0028 abc	0.0040 ± 0.0006 c	0.0031 ± 0.0035 ab	-
	LGG E DS	0.3728 ± 0.1153 b	0.0242 ± 0.0017 d	0.0035 ± 0.0001 c	0.0033 ± 0.0022 ab	-
	LGG NE DS	0.09835 ± 0.0085 d	0.0372 ± 0.0007 abc	0.0031 ± 0.0001 c	0.0047 ± 0.0002 ab	0.0001 ± 0.0002 b
	BY E S	0.2309 ± 0.0160 c	0.0413 ± 0.0057 a	0.0184 ± 0.0015 a	0.0021 ± 0.0011 b	0.0003 ± 0.0005 b
	BY NE S	0.1069 ± 0.0187 d	0.0381 ± 0.0033 ab	0.0183 ± 0.0020 a	0.0023 ± 0.0008 b	0.0014 ± 0.0014 a
	BY E DS	0.1085 ± 0.0085 d	0.0321 ± 0.0013 bc	0.0145 ± 0.0079 ab	0.0056 ± 0.0013 ab	-
	BY NE DS	0.0445 ± 0.0034 d	0.0319 ± 0.0005 bc	0.0101 ± 0.0003 bc	0.0081 ± 0.0018 a	-
15	LGG E S	0.9849 ± 0.0895 a	0.0343 ± 0.0022	0.0036 ± 0.0017 b	0.0042 ± 0.0013 b	-
	LGG NE S	0.3005 ± 0.0138 c	0.0345 ± 0.0012	0.0053 ± 0.0005 b	0.0041 ± 0.0028 b	0.0009 ± 0.0012
	LGG E DS	0.6260 ± 0.0208 b	0.0274 ± 0.0014	0.0037 ± 0.0001 b	0.0022 ± 0.00013 b	0.0001 ± 0.0003
	LGG NE DS	0.0425 ± 0.0190 d	0.0406 ± 0.0183	0.004 ± 0.0014 b	0.0048 ± 0.0006 ab	-
	BY E S	0.2252 ± 0.0082 c	0.0429 ± 0.0039	0.0179 ± 0.0012 a	0.0014 ± 0.0015 b	0.0001 ± 0.0002
	BY NE S	0.0959 ± 0.0345 d	0.0331 ± 0.0028	0.0125 ± 0.0080 ab	0.0029 ± 0.0011 b	0.0011 ± 0.0013
	BY E DS	0.1212 ± 0.0092 d	0.0332 ± 0.0012	0.0164 ± 0.0092 a	0.0039 ± 0.0017 b	-
	BY NE DS	0.0427 ± 0.0184 d	0.0278 ± 0.0048	0.0101 ± 0.0029 ab	0.0095 ± 0.0040 a	-
21	LGG E S	0.8771 ± 0.0434 a	0.0313 ± 0.0013	0.0020 ± 0.0006	0.0048 ± 0.0013 abc	-
	LGG NE S	0.2509 ± 0.0212 bc	0.0312 ± 0.0008	0.0034 ± 0.0001	0.0047 ± 0.0019 abc	0.0004 ± 0.0004
	LGG E DS	0.4647 ± 0.3052 b	0.0401 ± 0.0173	0.0113 ± 0.0092	0.0012 ± 0.0006 d	0.0006 ± 0.0006
	LGG NE DS	0.0778 ± 0.0357 c	0.0290 ± 0.0081	0.0024 ± 0.0008	0.0046 ± 0.0008 abcd	-
	BY E S	0.4443 ± 0.2250 b	0.0367 ± 0.0112	0.0104 ± 0.0086	0.0019 ± 0.0022 cd	0.0001 ± 0.0002
	BY NE S	0.0897 ± 0.0256 c	0.0288 ± 0.0058	0.0046 ± 0.0018	0.0066 ± 0.0013 ab	0.0040 ± 0.0080
	BY E DS	0.0882 ± 0.0261 c	0.0286 ± 0.0033	0.0120 ± 0.0072	0.0043 ± 0.0012 bcd	0.0021 ± 0.0042
	BY NE DS	0.0445 ± 0.0103 c	0.0279 ± 0.0023	0.0114 ± 0.0033	0.0078 ± 0.0018 a	0.0002 ± 0.0004

Content below the detection limit (0.0001). Factors: Nu-trish^®^ LGG^®^ (LGG), Nu-trish^®^ BY-01 DA (BY), with (E) and without (NE) addition of enzymes, and with (S) and without (DS) salt. Data are expressed as mean ± standard deviation (n = 8). Values in the same column of the same frame with different letters differ significantly (Tukey test; *p* < 0.05). No letters mean no significant differences.

## Data Availability

The original contributions presented in the study are included in the article, further inquiries can be directed to the corresponding authors.

## Appendix A

**Table A1:** Contents of glucose, maltose and isomaltose in the fermented drinks at 1, 15, and 21 days of storage. Factors: Nu-trish^®^ LGG^®^ (LGG), Nu-trish^®^ BY-01 DA (BY), with (E) and without (NE) addition of enzymes, and with (S) and without (DS) salt.

Fermentation Time (d)	Sample	Glucose (g per 100 g)	Isomaltose (g per 100 g)	Maltose (g per 100 g)
1	LGG E S	10.9632 ± 0.9499 a	0.0341 ± 0.0092 ab	0.0605 ± 0.0352 cd
	LGG NE S	0.0024 ± 0.0011 c	0.0145 ± 0.0004 cde	0.6304 ± 0.0120 a
	LGG E DS	10.7377 ± 1.5658 a	0.0244 ± 0.0005 bcd	0.1035 ± 0.0084 c
	LGG NE DS	0.0015 ± 0.0005 c	0.0059 ± 0.0005 de	0.2557 ± 0.0028 b
	BY E S	8.4065 ± 4.7617 ab	0.0295 ± 0.0030 bc	0.0364 ± 0.0372 cd
	BY NE S	0.0056 ± 0.0078 c	0.0010 ± 0.0018 e	0.0474 ± 0.0814 cd
	BY E DS	5.6437 ± 2.0498 b	0.0483 ± 0.0202 a	0.0606 ± 0.0173 cd
	BY NE DS	0.001415 ± 0.0028 c	-	0.0051 ± 0.0073 d
15	LGG E S	11.9013 ± 0.9913 a	0.0563 ± 0.0011 b	0.0162 ± 0.0011 d
	LGG NE S	0.0021 ± 0.0008 c	0.0139 ± 0.0009 c	0.6284 ± 0.0503 a
	LGG E DS	11.0114 ± 1.4064 ab	0.0459 ± 0.0073 b	0.0275 ± 0.0235 d
	LGG NE DS	0.0032 ± 0.0030 c	0.0059 ± 0.0006 c	0.2607 ± 0.0155 b
	BY E S	10.0508 ± 4.7908 ab	0.0524 ± 0.0011 b	0.0323 ± 0.0202 cd
	BY NE S	0.0055 ± 0.0006 c	0.0001 ± 0.0002 c	0.0092 ± 0.0106 d
	BY E DS	7.1052 ± 0.8121 b	0.0771 ± 0.0144 a	0.0831 ± 0.0099 c
	BY NE DS	-	-	0.0033 ± 0.0041 d
21	LGG E S	12.4819 ± 1.2800 a	0.0579 ± 0.0195 ab	0.0193 ± 0.0016 b
	LGG NE S	0.0010 ± 0.0003 b	0.0131 ± 0.0015 c	0.6075 ± 0.0657 a
	LGG E DS	12.8468 ± 1.8321 a	0.0601 ± 0.0231 a	0.0346 ± 0.0273 b
	LGG NE DS	0.0079 ± 0.0092 b	0.0092 ± 0.0045 c	0.4520 ± 0.2317 a
	BY E S	9.3650 ± 4.9087 a	0.0510 ± 0.0153 ab	0.0096 ± 0.0023 b
	BY NE S	0.0024 ± 0.0028 b	-	0.0057 ± 0.0046 b
	BY E DS	2.0348 ± 0.3807 b	0.0289 ± 0.0104 bc	0.0136 ± 0.0009 b
	BY NE DS	0.0013 ± 0.0025 b	-	0.0021 ± 0.0025 b
